# Supplementary material for: Firocoxib as a Potential Neoadjuvant Treatment in Canine Patients with Triple-Negative Mammary Gland Tumors
Source: Animals (Basel). 2022 Dec 23;13(1):60. doi: 10.3390/ani13010060 (PMC9817520; doi:10.3390/ani13010060)
Supplement: Supplementary file 1 [file animals-13-00060-s001.zip › animals-2065723-supplementary.pdf]

Table S1. Number of double stained cells for COX-2 and Caspase-3 in canine mammary gland tumors.

Identification	COX-2/Casp3 D0	COX-2/Casp3 D14
Case 1	12	15
Case2	8	11
Case 3	4	7
Case 4	7	9
Case 5	11	9
Case 6	9	10
Case 7	8	8
Case 8	11	10
Case 9	10	14
Case 10	4	8
Case 11	5	6
Case 12	6	7
Case 13	4	15
Case 14	7	19
Case 15	15	27
Case 16	11	28
Case 17	9	19
Case 18	6	21
Case 19	8	15
Case 20	10	11
Case 21	7	13
Case 22	8	17
Case 23	6	25
Case 24	12	16
Case 25	11	10
